# Molecular Profile of Phage Infection: A Novel Approach for the Characterization of *Erwinia* Phages through qPCR

**DOI:** 10.3390/ijms21020553

**Published:** 2020-01-15

**Authors:** Michael Parcey, Steven Gayder, Alan J. Castle, Antonet M. Svircev

**Affiliations:** 1 Centre for Biotechnology, Brock University, St. Catharines, ON L2S 3A1, Canada; mp17ll@brocku.ca (M.P.); sg10yl@brocku.ca (S.G.); 2 Agriculture and Agri-Food Canada, Vineland Station, ON L0R 2E0, Canada; 3 Department of Biological Sciences, Brock University, St. Catharines, ON L2S 3A1, Canada; acastle@brocku.ca

**Keywords:** bacteriophage, *Erwinia amylovora*, *Pantoea agglomerans*, biocontrol, adsorption, burst size, latent period

## Abstract

Due to the emergence of antibiotic resistance, phage-mediated biocontrol has become an attractive alternative for pathogen management in agriculture. While the infection characteristics of many phages can be adequately described using plaque assays and optical density, the results from phages of the apple pathogen *Erwinia amylovora* have low reproducibility with these techniques. Using quantitative real-time PCR (qPCR), the stage of the lytic cycle was determined through a combination of chloroform-based sampling, centrifugation, and DNase treatment. Monitoring the transition of phage genomes through the lytic cycle generates a molecular profile from which phage infection characteristics such as adsorption rate and burst size can be determined. To our knowledge, this is the first report of qPCR being used to determine these infection parameters. The characteristics of four different genera of *Erwinia* phages were determined. The phage ΦEa46-1-A1 was able to adsorb at a rate up to 6.6 times faster than ΦEa35-70 and ΦEa9-2. The low enrichment titer of ΦEa9-2 was shown to be due to the absence of lysis. The ΦEa46-1-A1 and ΦEa21-4 phages had the highest productivity, with burst sizes of 57 virions in 38 min and 185 virions in 98 min, respectively, suggesting these genera would make stronger candidates for the phage-mediated biocontrol of *E. amylovora*.

## 1. Introduction

*Erwinia amylovora* is a bacterial phytopathogen of rosaceous plants. Under optimal weather conditions, the pathogen has the capacity for complete infection of pome fruit orchards, leading to their destruction [1]. The fire-scorched appearance of the infected trees has given the disease its common name, fire blight. The antibiotic streptomycin is commonly used for the control of the *E. amylovora* during open bloom [2,3]. The large scale application of antibiotics has led to the selection and increased prevalence of streptomycin-resistant strains [4,5,6]. This development has driven researchers to re-examine the use of bacteriophages as biological control agents in orchard-integrated pest management practices [7,8,9,10].

Our phage-mediated biological control system utilizes naturally occurring *Erwinia* phages in combination with *Pantoea agglomerans,* a common orchard epiphyte that is susceptible to *Erwinia* phage infection [7,8,11]. In this system, *P. agglomerans*, referred to as the carrier, limits the growth of *E. amylovora* on the blossom through competitive exclusion [12]. *P. agglomerans* cells are also infected with *Erwinia* phages prior to application. On the blossom stigma, *P. agglomerans* acts as a phage reservoir, delivering and propagating the phage population [10]. To prevent the phage from inhibiting the carrier, these phages are applied at a low concentration. When the pathogen arrives, the phages have a limited period of time to suppress *E. amylovora* before ingress into the shoot and infection occurs [7,10]. This makes a phage’s adsorption rate, burst size, and time to lysis of particular interest in regards to *E. amylovora*. It is thought that through the synergistic use of *P. agglomerans* and *Erwinia* phages, a field efficacy comparable to antibiotics can be achieved [7].

To select phages for the biocontrol project, the phage-host interactions during infection need to be explored for both *P. agglomerans* and *E. amylovora*. The hosts used in this study for the propagation of each phage are considered “ideal hosts” and are selected based on the isolation host, efficiency of plating, and ability to produce high titers of the phage over 8 h [13,14]. Using these hosts, certain phage characteristics can be determined and compared under each phage’s ideal infection conditions. For over 50 years, optical density (OD_600_) measurements have been used for classically studied systems such as *Escherichia coli* to determine the timing and ability of the phages to lyse cells [15,16,17,18,19,20]. Quantitative measurements, required to define burst size and adsorption, commonly use plaque assays to quantify the production of viable phage progeny [16,21]. Within the *E. amylovora* and *P. agglomerans* systems, exopolysaccharides (EPS) interfere with optical density readings and also greatly impact plaque formation. This exacerbates the inconsistencies and limitations of both techniques [16,21,22,23,24].

**Figure 1:**
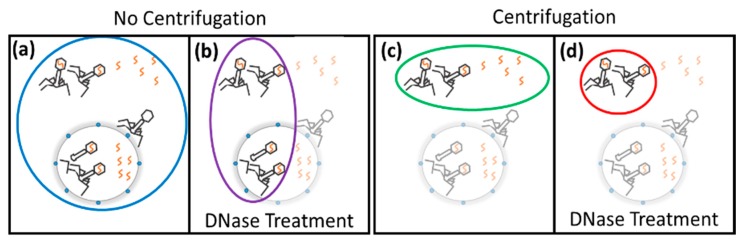
Modifying qPCR quantification to select for encapsidated and non-absorbed phage genomes. (**a**) Standard qPCR quantification will quantify all phage genomes and virions in solution. (**b**) DNase treatment selects only virions. (**c**) After centrifugation, the supernatant contains only non-adsorbed phage genomes and virions. (**d**) The use of centrifugation and DNase treatment selects non-adsorbed virions. The circles represent the selected genomes and the color of the circle corresponds to the curves of the MPI. Chloroform sampling is assumed to have been used for all qPCRs.

Molecular techniques, primarily quantitative real-time PCR (qPCR), have emerged as viable alternatives for the quantification of phages over the past 15 years [21,25,26,27]. Rudimentary qPCR techniques quantify the total number of genomic copies present (Figure 1a). Chloroform can be added to rupture the cells and the addition of DNase removes all non-encapsidated phage genomes. This method is used to quantify the number of virions (Figure 1b) [28,29,30]. Additionally, qPCR can be combined with the conventional method of determining the rate of adsorption and lysis through centrifugation. Quantifying the supernatant using qPCR determines the number of non-adsorbed phage genomes and virions present (Figure 1c). The use of both centrifugation and DNase treatment ensures only non-adsorbed virions are selected (Figure 1d) [31,32].

**Figure 2:**
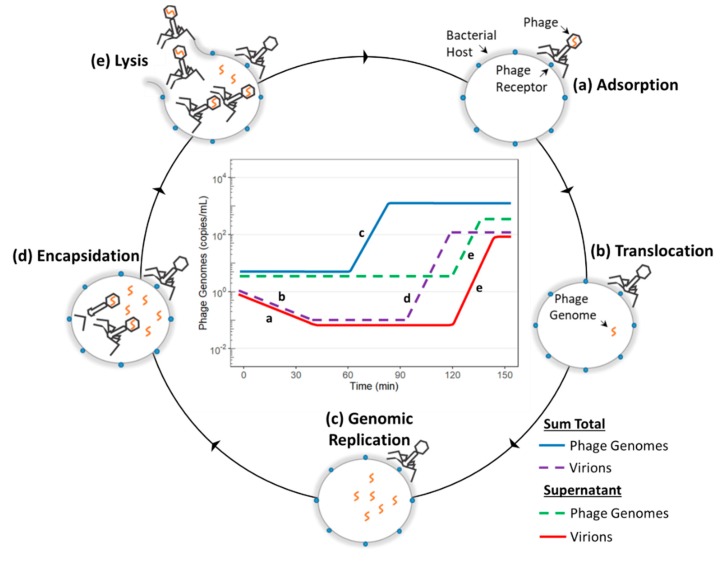
Transition states of the phage genome through the lytic cycle and their associated curves in a simulated molecular profile of phage infection. (**a**) Adsorption of the phage into the bacterial hosts leads to a decrease in the number of virions in the supernatant. (**b**) Ejection of the phage genome into the host leads to a decrease in the total number of phage particles. (**c**) Replication of the phage genome leads to an increase in the total number of phage genomes. (**d**) Expression of phage structural proteins leads to phage genome encapsidation and an increase in the overall number of virions. (**e**) Lysis leads to an increase in the number of virions and phage genomes in the supernatant.

When using qPCR to determine infection parameters, it is important to understand the major stages in the lytic phage lifecycle from a genomic perspective. The lytic cycle begins with the adsorption of the virion to the phage receptor located on the surface of the bacteria. As the phage bind to their hosts, there is a decrease in the number of virions present in the supernatant (Figure 1d, red and Figure 2a, red). When irreversible binding is achieved, the phage ejects its genome into the bacteria. As these translocated genomes are no longer protected by the phage capsid, they can be degraded by DNase. This leads to a decrease in the overall number of phage particles quantified and can be used to determine the percentage of phage that caused infection and the rate at which infection initiated (Figure 1b, purple and Figure 2b, purple). Once internalized, the phage genomes begin to replicate, increasing the total number of phage genomes present (Figure 1a, blue and Figure 2c, blue). Expression of the phage structural proteins leads to encapsidation of the phage genomes and increases the total number of phage particles (Figure 1b, purple and Figure 2d, purple). Finally, lysis of the bacterial host releases new phage particles (Figure 1d, red and Figure 1e, red) and non-encapsidated phage genomes (Figure 1c, green and Figure 1e, green) into the supernatant. The period of time from phage introduction to the lysis event is defined as the latent period, whereas the time from introduction to phage encapsidation is defined as the eclipse period.

In this study, four genera of *Erwinia* phages were characterized by qPCR to create a molecular profile of phage infection (MPI) on each phage’s ideal host. The *Myoviridae* phages ΦEa21-4 (*Kolesnikvirus Erwinia virus Ea214* [11]) and ΦEa35-70 (*Agricanvirus Erwinia virus Ea35-70* [33]), as well as the *Podoviridae* phage ΦEa9-2 (*Johnsonvirus Erwinia virus Ea9-2* [11]), were chosen as they are the type species of their genera. The *Podoviridae* ΦEa46-1-A1 [7] was also chosen for examination as it is a member of the separate *Zindervirus Erwinia virus Era103* [34] species, which rapidly reaches high titers when cultured. Each phage was characterized on either *E. amylovora* or *P. agglomerans*. While determining the host range of these *Erwinia* phages [13], ΦEa9-2 was noted to have an uncharacteristically high maximum titer in *E. amylovora* Ea273 (submitted as ATCC 49946) [35], and so the infection of both host isolates was explored. With all phage–host combinations a unique MPI was generated, from which the rate of adsorption/infection, time to genomic replication, eclipse period, latent period, and burst size of the phage population were determined.

## 2. Results

### 2.1. Correlation of Molecular Quantification of Plating Techniques

**Figure 3:**
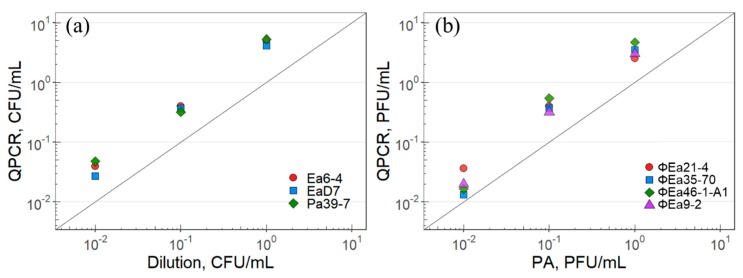
Correlation between the quantification using plating techniques and qPCR-based assays. (**a**) Bacterial quantification using dilution plating compared to qPCR for *E. amylovora* (Ea) and P. *agglomerans* (Pa). (**b**) Phage quantification using plaque assays compared to DNase-based qPCR for the four phages tested. The symbols represent the mean of matched-pair data produced in triplicate, which was normalized to the initial CFU/mL or PFU/mL. The diagonal lines represent prefect agreement between the two techniques.

The qPCR quantification of *E. amylovora* strains Ea6-4 and EaD7, as well as *P. agglomerans* Pa39-7 (carrier), was compared to the quantification through dilution plating in triplicate. The R^2^ values for the 9 replicates was greater than 0.98, except for one of the Pa39-7 replicates, which was 0.91. The quantification itself through qPCR was consistently higher than dilution plating by a factor of 2.7 to 5.3 (Figure 3a). In comparison, the R^2^ values of the 12 phage replicates showed a slightly weaker correlation, with 11 replicates having a R^2^ greater than 0.95 and one replicate of ΦEa21-4 showing only 0.88. Similar to the bacterial quantification, however, the qPCR results for phage ΦEa9-2, ΦEa21-4, ΦEa35-70, and ΦEa46-1-A1 were consistently higher than plaque assays by a factor of 1.3 to 5.4 (Figure 3b).

### 2.2. E. amylovora Infection by ΦEa21-4 and ΦEa46-1-A1

**Figure 4:**
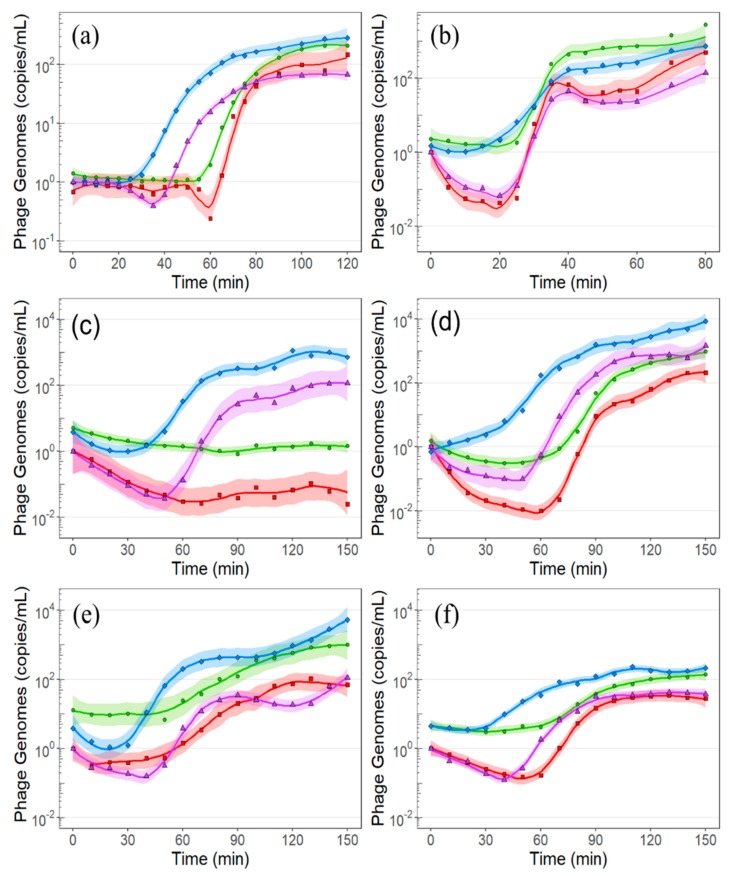
Molecular profile of phage infection of the four genera of *Erwinia* phages in this study. (**a**) Infection of *E. amylovora* Ea6-4 by phage ΦEa21-4 at an multiplicity of infection (MOI) of 0.45. (**b**) Infection of *E. amylovora* EaD7 by phage ΦEa46-1-A1 at an MOI of 0.625. (**c**) Infection of *E. amylovora* Ea17-1-1 by phage ΦEa9-2 at an MOI of 0.015. (**d**) Infection of *E. amylovora* Ea273 by phage ΦEa9-2 at an MOI of 0.015. (**e**) Infection of *P. agglomerans* Pa39-7 by phage ΦEa35-70 at an MOI of 0.03. (**f**) Infection of *P. agglomerans* Pa39-7 by phage ΦEa35-70 at an MOI of 0.7. The symbols represent the mean of data produced in triplicate, the line is a local regression model of the data (LOESS), and the shaded area around the line represents a 95% confidence interval. MOIs were calculated using the qPCR data at t_0_. Here, 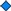 (blue diamond) = total number of phage genomes; 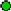 (green circle) = total number of phage genomes in the supernatant; 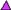 (purple triangle = total number of virions; 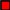 (red square = total number of virions in the supernatant.

Infection of *E. amylovora* Ea6-4 cells by phage ΦEa21-4 resulted in no observable decrease in the number of phage particles in the supernatant for the first 55 min (Figure 4a, red). Infection of the host cells began 18 min after phage introduction (Figure 4a, purple). During the eclipse period, only 60.7% of the phage population infected host cells (Table 1). The lytic cycle was completed after 98 min, producing a burst size of 185 virions. The *Podoviridae* phage ΦEa46-1-A1 had a distinct adsorption curve when infecting *E. amylovora* EaD7 over the first 10 min (Figure 4b, purple). This two-step adsorption pattern showed two adsorption rates when monitoring the quantity of phage particles in the supernatant: a rapid initial rate from 0 to 5 min, followed by a much slower secondary adsorption rate from 5 to 10 min. The distinguishing feature of ΦEa46-1-A1 was that there was no detectable difference between the eclipse period and latent period, with both initiated at 23 min (Figure 4b, red, purple). The lytic cycle was 38 min in length, with a burst size of 57 virions (Table 1).

### 2.3. E. amylovora Infection by Phage ΦEa9-2

*E. amylovora* Ea17-1-1 and Ea273 show two distinct adsorption patterns when interacting with phage ΦEa9-2. Attachment of ΦEa9-2 to Ea17-1-1 followed a first-order adsorption model, with a persistent adsorption constant over the first 60 min (Figure 4c, red). Contrarily, ΦEa9-2 bound to Ea273 following a two-step adsorption pattern, with a rate change after 20 min (Figure 4d, red). The number of phages that adsorbed to Ea17-1-1 was nearly equal to the number of phage that caused infection. In regard to Ea273, 99% of the phages adsorbed while only 90.3% caused infection (Table 1).

The maximum rate at which phage encapsidation occurred was conserved between Ea273 and Ea17-1-1, but this rate was maintained longer in Ea273, resulting in more phage progeny. The striking difference between the two hosts was that ΦEa9-2 was unable to induce a lytic burst in Ea17-1-1, resulting in a phage-laden state. The lytic cycle in Ea273 completed in 137 min, with a burst size of 850 virions. If lysis had occurred in Ea17-1-1, ΦEa9-2 would have had a burst size of 51 virions.

### 2.4. P. agglomerans Infection by Phage ΦEa35-70

The MPI was generated for phage ΦEa35-70 infecting *P. agglomerans* Pa39-7 at multiplicity of infection (MOI) of 0.03. The standard error of the mean was higher than expected, especially in the centrifuged samples (Figure 3e). The experiment was then repeated with an MOI of 0.7. Adsorption and infection by phage ΦEa35-70 began immediately after phage introduction for MOI_0.7_ (Figure 3f, red, purple) through first-order kinetics. Despite the difference in the MOI, there was no significant change in the overall percentage of phages that were able to cause infection (Table 1). Additionally, when MOI was accounted for, the burst size was conserved at 43 and 42 virions for MOI_0.7_ and MOI_0.03_, respectively.

## 3. Discussion

### 3.1. Molecular Quantification and Plating Techniques

The comparison of the quantification between traditional plating techniques and qPCR (Figure 3) for both bacteria and phages demonstrated that the molecular techniques were found to be consistently higher by a factor of up to 5.4. This is the expected result, as both species of bacterial hosts were quantified during exponential growth and more than one genomic copy could be present within a cell. Additionally, DNase cannot be used to remove extracellular bacterial genomes. In phages, the use of a DNase treatment in conjunction with qPCR allows for the absolute quantification of virions. Previous studies have shown qPCR quantification differs from plaque assays by a constant [21,28]. This is attributed to phage viability and the plaquing efficiency [21,30]. If the discrepancies between virion quantity and PFU/mL are due to viability, one would expect the percentage of virions that caused infection, and consequently the burst size, to be low. In the case of *Erwinia* phages, the majority of virions caused infection and the burst size for ΦEa21-4 was similar to *E. coli* phages, such as T4 [36]. It should be noted that the ideal hosts used in this study have no known resistance to these phages [37], and the phages themselves are non-lysogenic [27], eliminating these factors as potential causes for an observed decrease in viability. From this, one can conclude that plaquing using *Erwinia* phages is simply inefficient and does not represent the quantity of phage present.

### 3.2. Adsorption, Infection, and Genomic Replication

Many tailed-phages, such as those used in this study, adsorb to a bacterial host, rapidly interacting with the phage receptor through irreversible binding, and then translocate their genome into the host [38]. If the number of phage receptors far outnumber the quantity of phage, adsorption will occur at a constant exponential rate, observed through first-order kinetics [32]. This constant adsorption pattern was observed with *P. agglomerans* Pa39-7 when exposed to phage ΦEa35-70 at an MOI of 0.7 (Figure 4f, red), suggesting the phage receptor is well-expressed in Pa39-7. First-order adsorption was also observed in phage ΦEa9-2 infection of *E. amylovora* Ea17-1-1 (Figure 4c, red); however, this could be due to the decrease in MOI and may not reflect the phage receptor quantity.

When phage ΦEa21-4 was introduced to *E. amylovora* Ea6-4, there was no observable change in the number of phage particles in the supernatant until lysis (Figure 4a, red). Adsorption must have occurred for the subsequent infection and burst to have also occurred. The measurements for the centrifuged samples reported within a MPI are normalized to the initial number of phage particles in the supernatant. This means that all phages capable of adsorption must have done so prior to the initial measurement. It would also suggest either adsorption is incredibly fast or centrifuging the sample artificially increased the observed level of adsorption. In other words, centrifuging the phage may have caused them to adsorb to the host. While this result could suggest a very high affinity for the phage ΦEa21-4 to *E. amylovora*, further study would be required to determine the root cause of this phenomena.

In a two-step adsorption pattern, the phage will initially adsorb at a rapid rate, and then transition to a much slower rate [31,38]. This change in rate was observed after 20 min during the adsorption of phage ΦEa9-2 to *E. amylovora* Ea273 (Figure 4d, red) and after 5 min during the adsorption of phage ΦEa46-1-A1 to *E. amylovora* EaD7 (Figure 4b, red). In contrast, phage ΦEa9-2 adsorbed to *E. amylovora* 17-1-1 at a constant rate under the same conditions. This would indicate the adsorption model is host-dependent and is not an intrinsic factor of the phage. Similar observations have been made using the T4 phage, in that the rate and method of phage adsorption changes in the presence of different lipopolysaccharide antigens, as well as outer membrane protein C [39].

The rate at which phages initiate infection and the adsorption rate are generally considered synonymous, due to the sequential dependency of the two events [38] and the inherent nature of plaque assays to select for only infecting phage [40]. With ΦEa9-2, however, these results show it is possible for adsorption to occur without subsequent infection. While all ΦEa9-2 virions that adsorbed to Ea17-1-1 caused infection, only 90.3% of adsorbed ΦEa9-2 virions caused infection in Ea273. This shows that while the adsorption and infection events may be kinetically similar, both should be regarded independently, even when using plaque assays. Additionally, this would imply that there are two independent factors involved in the process: one controlling adsorption through reversible binding and the other initiating infection through irreversible binding [41].

After a phage genome is successfully translocated into the host, phage reproduction begins with the replication of the phage genome. Generally, the genomic replication rate was conserved across all phages (Table 1), with the exception of phage ΦEa35-70 at an MOI of 0.7. This was not expected, as all four phages have different genomic sizes and different phage-encoded DNA polymerases.

### 3.3. Latent Period, Burst Size, and Impact of MOI

The end of the lytic phage lifecycle is marked by the lysis of the bacterial host and the release of phage progeny. Interestingly, phage ΦEa9-2 could not reliably induce a lytic burst when infecting strain Ea17-1-1 but was able to induce lysis in Ea273. In our recent host range study of *Erwinia* phages [13], Ea17-1-1 was representative of the majority of *E. amylovora* isolates regarding phage production of ΦEa9-2. Isolate Ea273 was an exception to this observation within the host range and was the only host able to replicate phage ΦEa9-2 to a high titer. It is, therefore, reasonable to assume the inability of *E. amylovora* to produce high titers of ΦEa9-2 is due to a delay of the lytic event. The detection of lysis inhibition without knowing it exists is a unique benefit of the MPI analysis. If a phage-laden cell is sampled through plaque assay it would show no change from the stock phage concentration if directly plated or, if artificially lysed through chloroform, a completed lytic cycle [42]. Both results could be misleading if not performed simultaneously. Notably, the quantity of internal ΦEa9-2 virions produced still plateaued within Ea17-1-1 (Figure 4c, purple). This would support the theory that there is a resource or spatial constraint on the maximum burst size that can be produced in a cell [36,43]. The latent period would then only control burst size if it occurred before the maximum phage titer was reached [44,45].

MOI has a major impact on how phages interact with their hosts [32,46]. At high relative phage concentrations, there is an increased probability that multiple phages will attach to a single bacterial cell [16,32,47]. Previous research suggests that *P. agglomerans* can only be infected by *Erwinia* phages at high relative MOIs (greater than 0.1) [7]. Subsequently, an MPI was generated for ΦEa35-70 when infecting Pa39-7 at MOIs of 0.03 and 0.7. One of the most prominent differences between the two experiments is the amount of variability within measurements at the lower MOI value seen through the increased standard error of the mean (Figure 4e,f). While the rates of infection events changed, the timing and the burst size, once compensated for using a Poisson distribution, remained the same. This would suggest that the compound effect of sampling variability and growth of non-infected bacteria leads to higher variability outside periods of exponential growth of the phage, particularly when the samples are centrifuged. Simply put, MOI did not change how *P. agglomerans* was infected by phage ΦEa35-70, only how well the technique monitored the infection.

### 3.4. Erwinia Phages as Biocontrol Agents

Open pear and apple blossoms are susceptible to infection by *E. amylovora* until petal fall [48]. Therefore, biological control agents used to prevent blossom infections have to remain effective for 5–10 days. The phage carrier *P. agglomerans* prolongs the viability of phages on the blossom but requires the use of a lower initial phage titer. In the carrier–phage system, the critical characteristics for selecting phages as biocontrol agents are adsorption and the rate of phage production. Total adsorption needs to be high and the adsorption rate can be considered as a measure of the affinity of a phage particle to a specific host [49]. In systems where two or more potential hosts are present, the adsorption rate can theoretically be used to approximate the relative proportion of phages that will infect each host. Additionally, the faster a phage can reproduce within the pathogen and the larger the burst, the greater the suppression of the *E. amylovora* population. If the lytic cycle is rapid enough, the phage can even avoid phage resistance mechanisms [50]. These critical biocontrol characteristics would suggest that ΦEa21-4 and ΦEa46-1-A1 have the highest potential as biocontrol agents of *E. amylovora*. Both phages have a large burst size relative to the time it takes to complete the lytic cycle. Phage ΦEa46-1-A1 also has the fastest adsorption rate of the tested phage genera. In contrast, it was also shown that phage ΦEa9-2 would make a poor biocontrol agent based on its inability to reliably produce a lytic burst in some isolates, while ΦEa35-70 would also be unsuccessful due to its slow adsorption rate and low phage production over time.

### 3.5. Strengths and Limitations of MPI

There are several advantages to using molecular techniques to determine phage characteristics, other than the increase in throughput. The larger range of detection when using qPCR is able to monitor log_10_ changes in a phage population without requiring dilutions, and the ability to multiplex using qPCR quantification allows multiple phages and the bacterial host populations to be monitored simultaneously. Furthermore, the sampling size for characteristics such as burst size can be determined from the population instead of individual measures. Delbrück’s original work in 1945 demonstrated that the burst size of the T1 phage occurs in a right-skewed distribution, in which individual measures can differ by an order of magnitude [43]. It is, therefore, pertinent that a very large number of samples be taken to compensate for the substantial variability. Using molecular quantification, the burst size of each replicate is the average of a ~10^8^ individual bursts, which is representative of the mean across the population. The MPI technique itself is not without limitations. The technique is dependent of the use of chloroform, which excludes the use with chloroform-sensitive phages. Additionally, the bacterial population needs to be synchronized during logarithmic growth, which may make it difficult to study the interaction of phages with bacteria during the stationary growth phase.

## 4. Materials and Methods

### 4.1. Phage Propagation and Bacterial Growth Conditions

Host bacterial isolates were plated on Difco^TM^ nutrient agar (NA) (BD, Sparks, Maryland, USA) from frozen stock (Microbank^TM^, ProLab Diagnostics, Richmond Hill, ON, Canada), and then the phage were propagated as previously described [13]. Briefly, two bacterial cultures were prepared in Difco^TM^ nutrient broth (NB) (BD, Sparks, MD, USA): 0.9 mL at 10^8^ CFU/mL in a 2 mL microcentrifuge tube and 100 mL at 10^6^ CFU/mL in a 250 mL beveled flask. A 100 µL aliquot of phage stock solution (~10^8^ PFU/mL) was then added to the 0.9 mL culture. Both cultures were incubated at 27 °C (165 rpm) in a Innova 44 shaking incubator (New Brunswick Scientific, Edison, NJ, USA). After 4 h, the contents of the 2 mL tube were transferred into the beveled flask and incubation continued overnight. The phage solution was then treated with chloroform, centrifuged, and passed through a 0.22 µm filter (Millipore, Billerica, MA, USA). The resulting phage stocks were stored in amber vials (Wheaton Industries, Millville, NJ, USA) with 1 mL of chloroform at 4 °C. All phages and bacteria hosts used in this study can be found in Table 2.

### 4.2. Standardization of Dilution Plating to qPCR

A 100 mL bacterial suspension was generated at 10^6^ CFU/mL in NB from NA-cultured bacteria. This suspension was then grown at 27 °C (165 rpm) for 4 h. A 1 mL sample of the exponentially growing bacteria was serially diluted to ~10^3^ CFU/mL with NB and 100 µL was spread on a NA plate and incubated for 24 h at 27 °C. The 1 mL aliquots of the non-diluted bacterial sample, the 10^−1^ dilution, and the 10^−2^ dilution were then all transferred to amber vials containing 50 µL of chloroform. These samples were then quantified through a duplex qPCR, which detects *E. amylovora* and *P. agglomerans* using a plasmid standard [7,13]. Briefly, each qPCR reaction contained 2 µL of sample, 200 nM of each primer, and 100 nM of the probe in MBI EVOlution Probe qPCR mix (Montreal Biotech Inc., Montreal, QC, Canada). Reactions were performed in a Stratagene Mx3005P thermocycler (Agilent Technologies, Santa Clara, CA, USA) under the following conditions: 10 min at 95 °C followed by 40 cycles of 10 s at 95 °C and 45 s at 54 °C. All primers and probes used in this study can be found in Table 3. Previously, a plasmid was designed containing the amplicons of the *E. amylovora*, *P. agglomerans*, and the four phage qPCR primers. As the sequence of the plasmid is known, the plasmid concentration can be easily determined and diluted to create a standard curve for qPCR quantification. More information on the creation of plasmid standards can be found in Gayder et al. [13]. Experiments were completed in triplicate and all of the data for a given biological replicate was normalized to CFU/mL, as determined through dilution plating.

### 4.3. Standardization of Plaque Assay to qPCR

A new phage culture was generated and quantified through the standard plaque assay using a soft agar overlay method [7,13,16]. The plaque assay plates were then incubated for 24 h at 27 °C before enumeration. The dilution series used to generate the plaque assay was also quantified through qPCR. A portion of each phage dilution was first treated with a DNase protocol [28]. A 8 µL aliquot of phage solution was combined with 1 µL of 10× DNase buffer (B0303S, NEB, Ipswich, MA, USA) and 1 µL of 2000 U/mL DNase I (M0303S, NEB, Ipswich, MA, USA) in a 96-well plate. The plate was sealed and samples were incubated with the following program in a TC-512 thermal cycler (Techne, Stone, UK): 40 min at 37 °C, 20 min at 95 °C, hold at 10 °C. The DNase treated samples as well as the original phage samples were quantified through qPCR under the same conditions as *E. amylovora* using their respective primers and probes (Table 3). All of the data for a given biological replicate was then normalized to the PFU/mL, as determined through plaque assays.

### 4.4. Generating a Molecular Profile of Phage Infection

A 100 mL bacterial suspension was generated at 10^6^ CFU/mL from a plated culture using NB in a 250 mL beveled flask. This subculture was then incubated at 27 °C and 165 rpm for 4 h to create an exponentially growing bacterial culture. The full bacterial culture was then centrifuged at 8000× *g* for 15 min and the pellet was resuspended in 9.9 mL of NB in a 50 mL Falcon tube (Corning Life Sciences, Tewksbury, MA, USA), generating a bacterial suspension of ~10^8^ CFU/mL. This was done to create a homogenous bacterial culture to synchronize phage infection. The phage of interest was separately diluted in NB so that the addition of 100 µL of phage solution would give the desired MOI when added to the bacterial suspension. After the addition of the phage, the bacterial suspension continued to be incubated at 27 °C and 165 rpm. At t_0_ and every 5 or 10 min thereafter, two 200 µL samples were taken. One 200 µL sample was stored in an amber vial containing 200 µL NB and 50 µL chloroform, while the other 200 µL sample was centrifuged at 4 °C for 1 min at 16,000× *g*. The supernatant of this sample was then placed in an amber vial with 200 µL of NB and 50 µL of chloroform. An 8 µL aliquot of the chloroformed samples for each time point was treated using the DNase, as described in Section 4.2. In total, 4 samples were quantified through a phage–host duplex qPCR per time point: the sample, the supernatant, the sample after DNase treatment, and the supernatant after DNase treatment.

Unlike plaque-based techniques, the MOI used to generate an MPI can be determined after the addition of the phage using the qPCR results at t_0_. The target MOI of ΦEa9-2 was 0.01, the target MOIs of ΦEa35-70 were 0.5 and 0.05, and the target MOIs of ΦEa21-4 and 46-1-A1 were 0.5. All mentions of MOI within this study can be considered MOI_input_ (the quantity of input phage to the number of bacteria cells) [51].

### 4.5. Data Analysis

The qPCR data was first corrected for the dilutions that occurred over the course of the sampling protocol, as well as the DNase protocol. The data for both centrifuged samples for an individual replicate were then normalized to the initial number of phage particles detected in the supernatant. This normalization was then repeated on the non-centrifuged samples using the initial number of particles in the suspension as the denominator. In the case of phage ΦEa21-4, the centrifuged samples were normalized to the measurement at 5 min after the curve had stabilized. Using R statistical software [52], the data were then plotted graphically using the ggplot2 package [53] and both the geom_point and geom_smooth (span = 0.35) functions. The geometric means were also calculated for each category and time point.

The rates of exponential growth or loss for each phase of the lytic cycle were calculated using the geometric mean with the equation:

(1) $$y=ae^{kt}$$

where *k* is the observed rate constant. An event of the lytic cycle was calculated to begin at the time when the trend lines for the current and previous stage intersect.

The burst size of the phage was calculated based on the number of intracellular phage particles, with the assumption that all phage particles should be released at lysis. The burst size can be considered the quantity of virions produced through infection divided by the quantity of phages that caused infection, resulting in the following equation:

(2) $$\frac{Phage_{Output}}{Phage_{Input}\;\times \;Infection_{Percent}}$$

where *Infection_Percent_* is the percentage of phages that caused infection. If a phage adsorbs poorly to a host, the fraction of the non-infecting phage (1 − *Infection_Percent_*) can be subtracted from the *Phage_Output_* for a more accurate burst size calculation. At a higher MOI (0.1 to 1), the probability that multiple phages will infect a single bacterium becomes non-negligible, and therefore must be taken into account. The probability of multiple phage infections follows a Poisson distribution [32]:

(3) $$f_{m}=\frac{e^{-M}M^{m}}{m!}$$

where *f_m_* is the probability of a multiple infection event, *M* is the multiplicity of infection, and m is the number of phage involved in that infection. The number of phages that cause infection on a single cell has minimal effect on the burst size [47]. Therefore, the percentage of phages that caused primary infections can be expressed as:

(4) $$Infection_{Primary}=Infection_{Percent}\;x\overset{\infty }{\underset{m=1}{\sum }}\left(\frac{1}{m}\left(\frac{f_{m}}{1-f_{0}}\right)\right)$$

where *Infection_Primary_* is the percentage of phages that caused primary infections. The number of primary infections can be used as a more accurate correction factor for the *Phage_input_* (substituting *Infection_Percent_*) when determining burst size as the MOI approaches 1.

## 5. Conclusions

The practical application of a phage begins with its quantification and characterization. While researchers continue to investigate novel bacteria through metagenomics and culturomics, plaque assays are poorly suited for research using unconventional hosts. The development of an MPI using qPCR has been able to compensate for the incompatibility and inconsistency that *Erwinia* phages have shown using traditional phage techniques. This methodology is also the first to use qPCR to determine a phage’s adsorption rate, latent period, burst size, and the proportion of infectious particles [30]. These characteristics are useful determinants when considering phages as candidates for biocontrol. Additionally, it should be possible to modify this methodology to easily investigate other unconventional phages, such as the phages of thermophilic bacteria [54] and the gut phageome [55].

## Figures and Tables

**Table 1:** Infection characteristics of the lytic cycle as determined through the molecular profile of phage infection.

	Lytic Cycle Stage					
	Adsorption ^A^	Infection ^B^	Genomic Rep.	Encapsidation	Lysis	
Phage (Host: MOI)	Rate (min^−1^): Max (%)	Rate (min^−1^): Max (%)	Start (min): Rate (min^−1^)	Start (min): Rate (min^−1^): Virions ^C^	Start (min): Rate (min^−1^): Burst Size	End (min)
**ΦEa21-4** (Ea6-4: 0.45)	ND	0.052: 60.7	29: 0.166	37: 0.191: 63	57: 0.318: 185	98
**ΦEa46-1-A1** (EaD7: 0.625)	0.290: 95.7	0.221: 93.4	21: 0.173	23: 0.537: 45	23: 0.712: 57	38
**ΦEa9-2** (Ea17-1-1: 0.015)	0.071: 97.6	0.074: 96.3	39: 0.154	52: 0.196: 49	ND: ND: 51	ND
**ΦEa9-2** (Ea273: 0.015)	0.167: 99.0	0.152: 90.3	35: 0.139	50: 0.215: 768	65: 0.301: 850	137
**ΦEa35-70** (Pa39-7: 0.7)	0.044: 87.8	0.051: 87.7	28: 0.090	42: 0.181: 31	56: 0.174: 43	108
**ΦEa35-70** (Pa39-7: 0.03)	ND	0.074: 84.3	28: 0.174	43: 0.160: 35	52: 0.090: 42	116

ND—Not determined; ^A^ In all cases, adsorption began at t_0_; ^B^ The percentage and rate at which phage caused infection; ^C^ The number of intracellular virions prior to lytic burst.

**Table 2:** Phage and bacterial isolates used in this study.

Family	Strain	*Genus species*	Accession	Reference
***Myoviridae***	ΦEa21-4	*Kolesnikvirus Erwinia virus Ea214*	NC_011811.1	[11]
	ΦEa35-70	*Agricanvirus Erwinia virus Ea35-70*	NC_023557.1	[11]
***Podoviridae***	ΦEa46-1-A1	*Zindervirus Erwinia virus Era103*	Unpublished	[11]
	ΦEa9-2	*Johnsonvirus Erwinia virus Ea9-2*	KF806588.1	[11]
***Enterobacteriaceae***	Ea6-4	*Erwinia amylovora*	Unpublished	[13]
	EaD7	*Erwinia amylovora*	Unpublished	[13]
	Ea17-1-1	*Erwinia amylovora*	Unpublished	[13]
	Ea273	*Erwinia amylovora*	NC_013971.1	[35]
	Pa39-7	*Pantoea agglomerans*	Unpublished	[27]

**Table 3:** Primers and probes used for molecular quantification of phage and bacteria.

Target	Name	Sequences (5’-3’)	Reference
ΦEa21-4	END37-F	TTCAGCTTTAGCGGCTTCGAGA	[13]
	END37-R	AGCAAGCCCTTGAGGTAATGGA	
	END37-P	/56-ROXN/AGTCGGTACACCTGCAACGTCAAGAT/3IAbRQSp/	
ΦEa35-70	RDH311-F	TGGAAGGTCTTCTTCGAGAC	[9]
	RDH311-R	GACTACCTGGGGATGTTCAG	
	RDH311-P	/56-ROX/GACGGAAAAGATCACGGTACTCTT/3IAbRQSp/	
ΦEa46-1-A1	STS3-F	GACAAACAAGAACGCGGCAACTGA	[13]
	STS3-R	ATACCCAGCAAGGCGTCAACCTTA	
	STS3-P	/56-FAM/AGATGAAGTAGGTTATCTTCACAGTGCCCT/3BHQ_1/	
ΦEa9-2	N14-F	CATTGGGTAATCCCTTTGAG	[13]
	N14-R	GATAGACTGGTTCCCCTGTG	
	N14-P	/56-FAM/TCTGGTGGA/ZEN/CAGAGACGATGTAAT/3IABkFQ/	
*E. amylovora*	Pa-Gnd-F	TGGATGAAGCAGCGAACA	[7]
	Pa-Gnd-R	GACAGAGGTTCGCCGAGA	
	Pa-Gnd-P	/5HEX/AAATGGACCAGCCAGAGCTCACTG/3BHQ-1/	
*P. agglomerans*	Ea-Lsc-F	CGCTAACAGCAGATCGCA	[7]
	Ea-Lsc-R	AAATACGCGCACGACCAT	
	Ea-Lsc-P	/5Cy5/CTGATAATCCGCAATTCCAGGATG/3IAbRQsp/	
